# On the Correlation of Physical Forces

**Published:** 1847-04-01

**Authors:** 


On the Correlation of Physical Forces. By *W. R. Grove*, Esq. M.A., F.R.S., Barrister at Law. 8vo. pp. 50. Highley, 1846.

The present Essay is the substance of a course of Lectures delivered by Mr. Grove at the London Institution, and most properly published at the request of the Proprietors. We believe we are correct in saying that the views herein unfolded have met with the acceptation of some of the leading observers in physical and natural science; and to our own minds the reasoning of the author carries conviction.

After adverting to the impossibility of our correctly applying the term cause otherwise than in a mere secondary sense, as indicating antecedent forces, it being predicable of no physical agency that it is abstractedly the cause of another, the author thus states the object he has in view:—

"The position which I seek to establish in this Essay is, that the various imponderable agencies, or the affections of matter which constitute the main objects of experimental physics, viz., Heat, Light, Electricity, Magnetism, Chemical Affinity, and Motion are all Correlative, or have a reciprocal dependence. That neither, taken abstractedly, can be said to be the essential or proximate cause of the others, but that either may, as a force, produce or be convertible into the other; thus heat may mediately or immediately produce electricity, electricity may produce heat; and so of the rest.

"The term Force, although used in very different senses by different authors, in its limited sense may be defined as that which produces or resists Motion. Although strongly inclined to believe that the five other affections of matter, which I have above named, are, and will ultimately be resolved into, modes of motion, it would be going too far, at present, to assume their identity with it; I therefore use the term Force, in reference to them, as meaning that active principle inseparable from matter, which induces its various changes."

In pursuance of this idea, the author enters into a detailed demonstration, which the facts already acquired by science enable him to do, that each of these affections or properties of matter is convertible into the five others. Thus, in respect to *Motion*, bodies impinging on each other, according as they may be homogeneous or heterogeneous, elicit *heat* or *electricity*. Heat and Electricity so produced will produce *magnetism*. *Light*, again, may be produced directly during friction, or mediately by means of electricity. *Chemical affinity* too, will result from electricity, which has initiated in motion. "Lastly, motion may again be reproduced by the forces which have emanated from motion: thus, the divergence of the electrometer, the revolution of the electrical wheel, the deflection of the magnetic needle, are palpable movements reproduced by the intermediate modes of force, which have themselves been originated in motion." So, too, if Heat, Light, Electricity, Magnetism, or Chemical Affinity, be taken as the starting point, the whole series may be induced. We regret that our limits prevent our following this demonstration in detail, as it is well deserving of our doing, especially as there is incidentally introduced some important speculations upon the non-existence of latent heat. We, however, cordially recommend the work to the notice of our readers as the ablest attempt of our time to simplify our views of physical science; and content ourselves with quoting a few of the concluding observations.

"The sense I have attached to the word correlation, in treating of physical phenomena, will, I think, be evident, from the previous parts of this essay, to be that of a reciprocal production or convertibility; in other words, that any force capable of producing or being convertible into another may, in its turn, be produced by it,—nay, more, can be itself resisted by the force it produces, in proportion to the energy of such production, as action is ever accompanied and resisted by reaction; thus, the action of an electro-magnetic machine is reacted upon by the magneto-electricity developed by its action.

"The evolution of one force or mode of force into another, has induced many to regard all the different natural agencies as reducible to unity, and as resulting from one force which is the efficient cause of all the others: thus, one party writes to prove that electricity is the cause of every change in matter; another that chemical action is the cause of everything; another that heat is the universal cause,—and so on. If, as I have submitted to you, the true expression of the fact is, that each mode of force is capable of producing the other, then any view which regards either of them as abstractedly the efficient cause of all the rest is erroneous; the view has, I believe, arisen from a confusion between the abstract or generalised meaning of the term cause, and its concrete or special sense,—the word itself being indiscriminately used in both these senses.

"Another confusion of terms has arisen, and has, indeed, much embarrassed me in enunciating the propositions sought to be proved in these pages, on account of the imperfection of scientific language; an imperfection in great measure unavoidable, it is true, but not the less embarrassing.

"Thus, the words light, heat, electricity, and magnetism, are constantly used in two senses, viz., that of the force producing, or the subjective idea of force or power, and of the effect produced, or the objective phenomenon. The word motion, indeed, is only applied to the effect, and not to the force,—and chemical affinity is generally applied to the force, and not to the effect; but the other four terms are applied indiscriminately to both, for want of a distinct terminology."

